# Sodium–glucose cotransporter 2 inhibitor Dapagliflozin attenuates diabetic cardiomyopathy

**DOI:** 10.1186/s12933-019-0980-4

**Published:** 2020-01-10

**Authors:** M. Arow, M. Waldman, D. Yadin, V. Nudelman, A. Shainberg, N. G. Abraham, D. Freimark, R. Kornowski, D. Aravot, E. Hochhauser, M. Arad

**Affiliations:** 1 Cardiac Research Laboratory, Felsenstein Medical Research Institute Petah-Tikva, Sackler Faculty of Medicine, Tel Aviv University, Tel Aviv, Israel; 2 Leviev Heart Center, Sheba Medical Center, Tel Hashomer and Sackler School of Medicine, Tel Aviv University, Tel Aviv, Israel; 3 Bar Ilan University, Ramat Gan, Israel; 4 Pharmacology Department, New York Medical College, Valhalla, NY 10595 USA

**Keywords:** Diabetes mellitus type 2, Cardiomyopathy, Dapagliflozin, Cardiomyocytes, Calcium transport fibrosis, Inflammation, ROS

## Abstract

**Background:**

Diabetes mellitus type 2 (DM2) is a risk factor for developing heart failure but there is no specific therapy for diabetic heart disease. Sodium glucose transporter 2 inhibitors (SGLT2I) are recently developed diabetic drugs that primarily work on the kidney. Clinical data describing the cardiovascular benefits of SGLT2Is highlight the potential therapeutic benefit of these drugs in the prevention of cardiovascular events and heart failure. However, the underlying mechanism of protection remains unclear. We investigated the effect of Dapagliflozin—SGLT2I, on diabetic cardiomyopathy in a mouse model of DM2.

**Methods:**

Cardiomyopathy was induced in diabetic mice (db/db) by subcutaneous infusion of angiotensin II (ATII) for 30 days using an osmotic pump. Dapagliflozin (1.5 mg/kg/day) was administered concomitantly in drinking water. Male homozygous, 12–14 weeks old WT or db/db mice (n = 4–8/group), were used for the experiments. Isolated cardiomyocytes were exposed to glucose (17.5–33 mM) and treated with Dapagliflozin in vitro. Intracellular calcium transients were measured using a fluorescent indicator indo-1.

**Results:**

Angiotensin II infusion induced cardiomyopathy in db/db mice, manifested by cardiac hypertrophy, myocardial fibrosis and inflammation (TNFα, TLR4). Dapagliflozin decreased blood glucose (874 ± 111 to 556 ± 57 mg/dl, p < 0.05). In addition it attenuated fibrosis and inflammation and increased the left ventricular fractional shortening in ATII treated db/db mice. In isolated cardiomyocytes Dapagliflozin decreased intracellular calcium transients, inflammation and ROS production. Finally, voltage-dependent L-type calcium channel (CACNA1C), the sodium–calcium exchanger (NCX) and the sodium–hydrogen exchanger 1 (NHE) membrane transporters expression was reduced following Dapagliflozin treatment.

**Conclusion:**

Dapagliflozin was cardioprotective in ATII-stressed diabetic mice. It reduced oxygen radicals, as well the activity of membrane channels related to calcium transport. The cardioprotective effect manifested by decreased fibrosis, reduced inflammation and improved systolic function. The clinical implication of our results suggest a novel pharmacologic approach for the treatment of diabetic cardiomyopathy through modulation of ion homeostasis.

## Background

Patients with diabetes are two to three times more likely to develop cardiovascular disease and are at an increased risk of having a myocardial infarction, stroke or develop heart failure [1, 2]. Once cardiovascular disease has developed, diabetic patients have a significantly worse prognosis compared to non-diabetic ones. Diabetes, obesity, insulin resistance and impaired glucose tolerance are associated with increased intra-myocardial levels of lipid which results in lipotoxicity, diastolic dysfunction, cardiac electrical disturbances and Ca^2+^ dysregulation [3, 4]. Long-term exposure to oxidative stress in diabetes mellitus induces chronic inflammation and fibrosis [5]. The expression of TNF-α and NF-κB along with collagen were elevated and accompanied by an increase in oxidative stress in the diabetic heart [6].

The diabetic heart is particularly susceptible to perturbations of angiotensin II (ATII). ATII, the effector peptide of the renin–angiotensin system, regulates volume and electrolyte homeostasis and is involved in cardiac and vascular growth. ATII elevates blood pressure (BP) through vasoconstriction, sympathetic nervous stimulation, increased aldosterone biosynthesis and renal action [2]. ATII promotes cardiac hypertrophy, inflammation, and fibrosis [7].

There is no established therapy for diabetic heart disease. Classical glucose-lowering therapies reduced the number of coronary events but generally had a neutral effect on cardiovascular mortality and sometimes resulted in heart failure exacerbation [8]. Sodium glucose cotransporter 2 inhibitors (SGLT2I) are a new class of drugs that have been recently FDA-approved for use to lower blood glucose in adults with DM2 given as either monotherapy or as add-on therapy. These drugs have been given priority in treating diabetes in patients with cardiovascular disease [9].

In 2015 a clinical trial with empagliflozin (EMPA-REG) reported a significant reduction of cardiovascular events and mortality in high risk diabetics on therapy [10]. A study on the effect Dapagliflozin (DAPA) on cardiovascular events (DECLARE-TIMI 58) reported a lower rate of both cardiovascular death or heart failure (HF) hospitalizations after treating high-risk diabetes [11]. Specifically, HF hospitalizations were reduced in all study population while mortality was affected only in those with history of heart failure due to reduced ejection fraction [12]. Importantly, these drugs improve the outcome of heart failure with reduced systolic function and accomplish that independently of glucose control [13].

SGLT2 antagonists act directly on the proximal tubule in the kidney, by blocking the sodium glucose transporter and decreasing renal glucose reabsorption they increase urinary glucose excretion [14]. Beyond glucose control, the mechanisms underlying the cardioprotective effects of SGLT2 inhibitors among patients with DM2 remain unclear. The primary isoform expressed in the heart is SGLT1 and little evidence exists for the expression of SGLT2 receptor in cardiac muscle [14]. It was suggested that these drugs have an indirect effect on the heart through amelioration of volume overload, lowering of blood pressure, weight reduction and improved renal hemodynamics [15].

The purpose of this study was to investigate the effect of SGLT2I- DAPA on the diabetic heart in a mouse model of DM2.

## Methods

### Animal studies

The animal experiments were approved by the institutional animal care and use committee of Tel Aviv University (01-18-001). We used male mice with leptin receptor deficiencies (db/db mice) that develop obesity and DM2 as an animal model. The genetic status of the mice (either heterozygous for the leptin receptor mutation or homozygous for the leptin receptor WT state or mutation) was determined by PCR. Male, homozygous db/db mice (C57BLKS/J-lepr^db^/lepr^db^) and their wild type (WT) littermates were maintained in a pathogen free facility on regular rodent chow with free access to water and 12-h light and dark cycles. These mice develop cardiomyopathy, when exposed to ATII stress. Cardiomyopathy is not found to a significant extent in the unstressed db/db hearts [16–18]. Male homozygous WT or db/db mice (n = 4−8/group), 12–14 weeks old, were used for the experiments.

#### Procedures

Cardiomyopathy was induced by a 4-week infusion of ATII using a subcutaneous pump. SGLT2I (DAPA) or vehicle was administered concomitantly in drinking water with ATII and compared with correspondent groups.

The following groups were studied:

1. WT mice + saline infusion for 1 month.
2. WT mice + ATII infusion for 1 month.
3. Db/db mice + saline infusion for 1 month.
4. Db/db mice + saline infusion + DAPA for 1 month.
5. Db/db mice + ATII infusion for 1 month.
6. Db/db mice + ATII infusion + DAPA for 1 month.

Angiotensin II: 12–14 week-old male mice were anesthetized with 2% isoflurane and an ALZET osmotic pump (Durect Corp., Cupertino, CA, USA) was subcutaneously implanted into each mouse. The osmotic pumps infused angiotensin II (Sigma-Aldrich, St. Louis, MO, USA) at a rate of 1000 ng kg^−1^ min^−1^ for 4 weeks [16].

Dapagliflozin: DAPA (ASTRAZENECA, DE 19850-5437 USA) was dissolved in drinking water. Dapagliflozin is soluble at 0.5 mg/ml in sterile water. The dosing solution for DAPA is light sensitive. The stock dosing solution needs to be prepared every week and stored in 4 °C protected from light. Three month old mice were treated with 1.5 mg/kg/day DAPA for 1 month [27]. In cell culture DAPA was dissolved in PBS in a final concentration of 5 μΜ (a clinically relevant concentration) [19].

Two-dimensional (2D) guided M-mode echocardiography was performed using an echocardiogram (Vevo 2100 Imaging System, VisualSonics, Toronto, Ontario, Canada) equipped with a 30-MHz linear transducer. Animals were lightly anesthetized by inhaling isoflurane. The 2D mode in the parasternal long-axis view is used to monitor the heart. From this view, an M mode cursor is positioned perpendicular to the interventricular septum and posterior wall of the left ventricle (LV) at the level of the papillary muscles. An M mode image was obtained at a sweep speed of 100 mm/s. Left-ventricular anterior and posterior wall thickness, end-diastolic dimensions (LVEDD), and left-ventricular end-systolic chamber dimensions (LVESD) were measured. The left-ventricular fractional shortening (FS) is calculated as [(LVDD − LVESD)/LVEDD] × 100 [16].

Systolic blood pressure was measured at the end of the experiment in awaken mice using a noninvasive computerized tail-cuff system (Blood pressure pump, Life science instrument, CA, USA). Mice were placed in temperature-controlled chambers (37 °C) and blood pressure is recorded in 2–3 cycles of 10 measurements.

#### Histopathology

Midventricular heart sections were fixed in 4% formalin, and then embedded in paraffin. Several transverse sections were cut from the paraffin-embedded samples and stained with hematoxylin and eosin. Sections from each heart were also stained with Masson trichrome for collagen deposition. The fibrotic area (3 mice per group, 10× fields each) was measured with the use of ImagePro PLUS software (Media Cybernetics, USA) [16].

Because hydroxyproline is found almost exclusively in the protein collagen the hydroxyproline has been used as a marker to quantify levels of collagen. Total hydroxyproline from heart tissue was determined with the Cell Biolabs’ Hydroxyproline Assay Kit [16].

#### Cell culture

Neonatal rat hearts (Sprague-Dawley 1–2 days old) were removed under sterile conditions and washed three times in phosphate-buffered saline (PBS) to remove excess blood cells. The hearts were minced and then gently agitated in a solution of proteolytic enzymes—RDB (Biological Institute, Ness-Ziona, Israel), which is prepared from fig tree extract. RDB is diluted 1:100 in Ca^2+^ and Mg^2+^-free PBS for a few cycles of 10 min each. Dulbecco’s modified Eagle’s medium (DMEM, Biological Industries, Kibbutz Beit Haemek, Israel) containing 10% horse serum was added to supernatant suspensions containing dissociated cells. The mixture was centrifuged at 300*g* for 5 min. The supernatant was discarded, and the cells were resuspended. The suspension of the cells was diluted to 1 × 10^6^ cells/ml, and 1.5 ml of the suspension was placed in 35-mm plastic culture dishes, or 0.4 ml in 24 wells plates [16]. The cultures were incubated in a humidified atmosphere of 5% CO_2_ and 95% air at 37 °C. Confluent monolayers exhibiting spontaneous contractions develop in culture within 2 days. DAPA (5 μM) was added to the cultures for 2 h ATII (1 µM) was added, than for another 2 h before analysis.

Calcium transient measurements in cardiomyocytes were conducted using the indicator indo-1-AM under a Zeiss epi-fluorescent inverted microscope. Cardiomyocytes grown on a coverglass, in 33 mM or 17.5 mM glucose, were incubated with 3 µM indo-1-AM and 1.5 μM pluronic acid for 30 min at 25 °C. After incubation, the cells were rinsed twice with glucose-enriched PBS and transferred to a chamber on the microscope. Indo-1 loaded cells were excited at 355 nm and the emitted light then split by a dichroic mirror into two photomultipliers (Hamamatsu, Japan), with input filters at 410 and 490 nm for indo-1. The fluorescence ratio (R) of 410 nm/490 nm, which was proportional to [Ca^2+^]c, was implemented to the Caplan program. Cells grown on coverslips were treated with ATII (1 μM) for 2 h and then DAPA (5 µM) was added. Calcium transient amplitude (AMP) and the time integral of Ca^2+^ transient was determined as the area under the curve (AUC) via the Caplan program, which gives the integral during any specified time window. The time window was the same for each experiment.

Oxidative stress was measured in cultured rat neonatal cardiomyocytes exposed to high or normal glucose concentration (33 mM or 17.5 mM) using a 2′,7′-dichlorofluorescin diacetate (DCF-DA) reagent (Sigma-Aldrich, St. Louis, MO, USA). This compound is an uncharged cell-permeable molecule. Inside cells, this probe is cleaved by nonspecific esterases, forming carboxydichlorofluorescein, which is oxidized in the presence of ROS. Cardiomyocytes were incubated with DAPA for 2 h; ATII was added to the cells and stand for another 2 h than the cells were loaded with 10 µM DCF-DA for 30 min at 37 °C [18].

#### Western blot analysis

Frozen-kept cardiac tissue samples (20 mg) were homogenized in lysis buffer and quantified for protein levels using a commercial assay (Bio-Rad, Israel). Protein (30–60 μg/lane) was separated on 10% SDS-polyacrylamide gel under denaturing conditions and electro blotted to a nitrocellulose membrane. The membrane was blocked by incubation for 2 h in 5% nonfat milk in Tris buffer containing 0.05% Tween-20 and then immunoblotted overnight at 4 °C with primary antibody. The secondary antibody was horseradish peroxidase-conjugated antibody. Expression was detected with ECL-detection kit (Santa Cruz Biotechnology, Dallas, TX, USA) [29]. Primary antibodies: β-actin (C4):sc-47778 (SANTA CRUZ Biotechnology, Inc Europe), NCX-1 (Cell Signaling technology, U.S.), NHE (Abcam, England) Anti-CaV1.2 (CACNA1C) antibody (Alomone labs Israel). Secondary antibodies: Goat Anti-Rabbit IRDye 680/800 LI-COR Biosciences, Goat Anti-Mouse IRDye 680/800 LI-COR Biosciences [18].

#### RT-PCR

Total RNA was purified from the hearts by using TRIzol (Ambion, Austin, TX, USA) as per manufacturer’s instructions. The quantity of total RNA was determined by OD260 measurements. cDNA was synthesized from total RNA using the SYBR green Kit according to the manufacturer’s protocol. Quantitative real-time PCR analysis was performed using the Step One Plus system (Applied Biosystems, Foster City, CA, USA) [16]. Relative mRNA level of the different genes (described in the tables below) was normalized to the level of the housekeeping gene RPL13a.

Primers sequences:

**Table:** 

Mice	S	AS
RPL13a	GCTTCTTCTTCCGATAGTGCATC	AGCCTACCAGAAAGTTTGCTTAC
Cola1	AATGGTGCTCCTGGTATTGC	GGTCCTCGTTTTCCTTCTT
TNFα	CAGGCTTGTCACTCGAATTTTG	CTTCTGTCTACTGAACTTCGGG
IL-1β	TTCTCCACAGCCACAATGAG	ACGGACCCCAAAAGATGAAG
TLR4	TCCCTGCATAGAGGTAGTTCC	TCCAGCCACTGAAGTTCTGA

Rats (cardiomyocytes):

**Table:** 

	S	AS
RPL13a	GGCAGGTTCTAGTATTGGATGG	CAGAAATGTTGATGCCCTCAC
TNFα	CTTCTCATTCCTGCTCGTGG	TGATCTGAGTGTGAGGGTCTG
IL-1β	TGCAGGCTTCGAGATGAAC	GGGATTTTGTCGTTGCTTGTC

### Statistical analysis

Results were expressed as mean ± standard deviation (SD). The statistical difference between two groups was assessed using a two tailed Student’s t test. To compare more than two groups we use the analysis of variance (ANOVA) with Duncan’s multiple comparison. Categorical values are compared using the Chi square or Fisher’s exact test, as appropriate. p < 0.05, considered significant.

## Results

### Animal model

#### DAPA attenuates metabolic dysfunction and diabetic cardiomyopathy

Diabetic (db/db) mice had elevated body weight, increased glucose level but normal heart weight, glucose and cholesterol blood levels when compared to WT (control) animals (Table 1). Diabetic mice had normal cardiac dimensions and function (Table 2) which were not significantly different from WT mice. ATII significantly (p < 0.05) increased blood pressure (44%) in both WT and diabetic mice but had no effect on blood glucose (Table 1). ATII treatment of WT and db/db mice resulted in wall thickening, reduced LV diastolic dimension, and reduced fractional shortening (Table 2). DAPA lowered blood glucose in db/db mice (Table 1). In ATII treated diabetic mice DAPA reduced blood pressure (p < 0.0002) and significantly improved the fractional shortening (Table 2). DAPA had no significant hemodynamic effect in db/db mice in the absence of ATII stress.

**Table 1:** Body and heart weight and biochemical markers

Groups/mice	Wt/wt	Wt/wt + ATII	Db/db	Db/db + DAPA	Db/db + ATII	Db/db + ATII + DAPA
Numbers	8	6	5	4	8	5
Body weight (g)	25.5 ± 1	25.5 ± 1.5	41.5 ± 5^+^	42.8 ± 2^+^	38.8 ± 8^+^	45.3 ± 5^+^
Blood pressure (mmHg)	95 ± 5	137 ± 5^+^	102 ± 3	97 ± 3	147 ± 3^+,^*	133 ± 2^+,^*^,#^
Blood glucose (mg/dl)	298 ± 55	258 ± 76	937 ± 75^+^	607 ± 30^+,^*	874 ± 111^+^	556 ± 57^+,&^
Blood cholesterol	118 ± 25	143 ± 5	120 ± 10	143 ± 22	202 ± 77	188 ± 35

^+^p < 0.05 vs wt/wt, *p < 0.05 vs *db/db*, ^&^p < 0.05 vs *db/db* + ATII, ^#^p < 0.002 vs *db/db* + ATII

**Table 2:** Heart dimensions and function

	Wt/wt	Wt/wt + ATII	Db/db	Db/db + DAPA	Db/db + ATII	Db/db + ATII + DAPA
Numbers	6	6	5	4	6	6
IVS	0.9 ± 0.1	1.2 ± 0.08^&^	0.96 ± 0.08	0.97 ± 0.1	1.2 ± 0.1^#^	1.2 ± 0.2
LVPW	0.8 ± 0.05	1.1 ± 0.12^&^	0.9 ± 0.1	0.9 ± 0.3	1.1 ± 0.1^#^	1.2 ± 0.3
LVEDD	3.8 ± 0.2	3.4 ± 0.3	3.5 ± 0.5	3.7 ± 0.6	3.7 ± 0.4	3.4 ± 0.6
LVESD	2.4 ± 0.1	2.3 ± 0.3	2.1 ± 0.5	2.2 ± 0.5	2.4 ± 0.3	1.9 ± 0.5*
FS	36.2 ± 2.2	31.9 ± 4.9	39.4 ± 6.2	39.7 ± 6.3	34.9 ± 2.6	45.9 ± 6.5*

* p < 0.05 vs *db/db* ± ATII, ^#^p < 0.05 vs *db/db*, ^&^p < 0.05 vs wt/wt

#### DAPA attenuates fibrosis and inflammation in diabetic cardiomyopathy

**Fig. 1:**
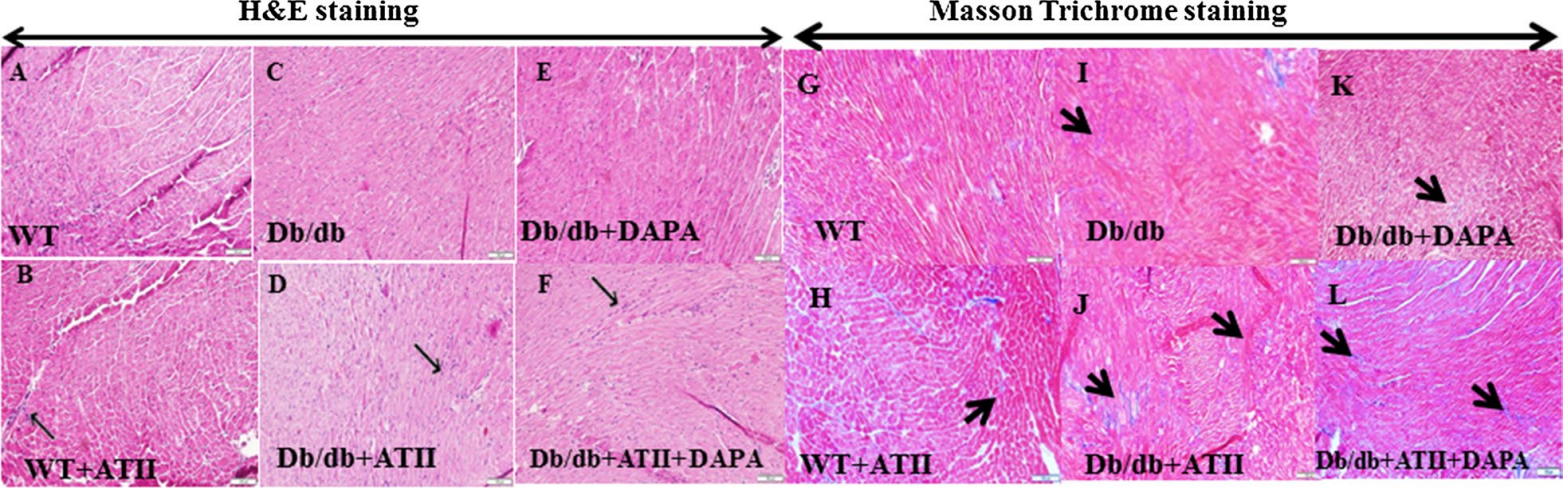
H&E staining: wt/wt and db/db mice showed normal architecture with very well-shaped cardiomyocytes in the heart (**a** and **c**). ATII treatment increased the mononuclear inflammatory cells infiltration in left ventricle of heart (**b** and **d**, thin arrows). DAPA ameliorated the damage as seen by the organized architecture of the cardiomyocytes and reduction of mononuclear inflammatory cells (**e** and **f**), Scale bar = 100 µm. Masson trichrome staining: ATII induced fibrosis (blue staining-thick arrows) in both wt/wt and db/db mice (**h** vs **g** and **j** vs **i**). Tissues treated with DAPA were stained with normal pink color (**l** and **k**), Scale bar = 100 µm

**Fig. 2:**
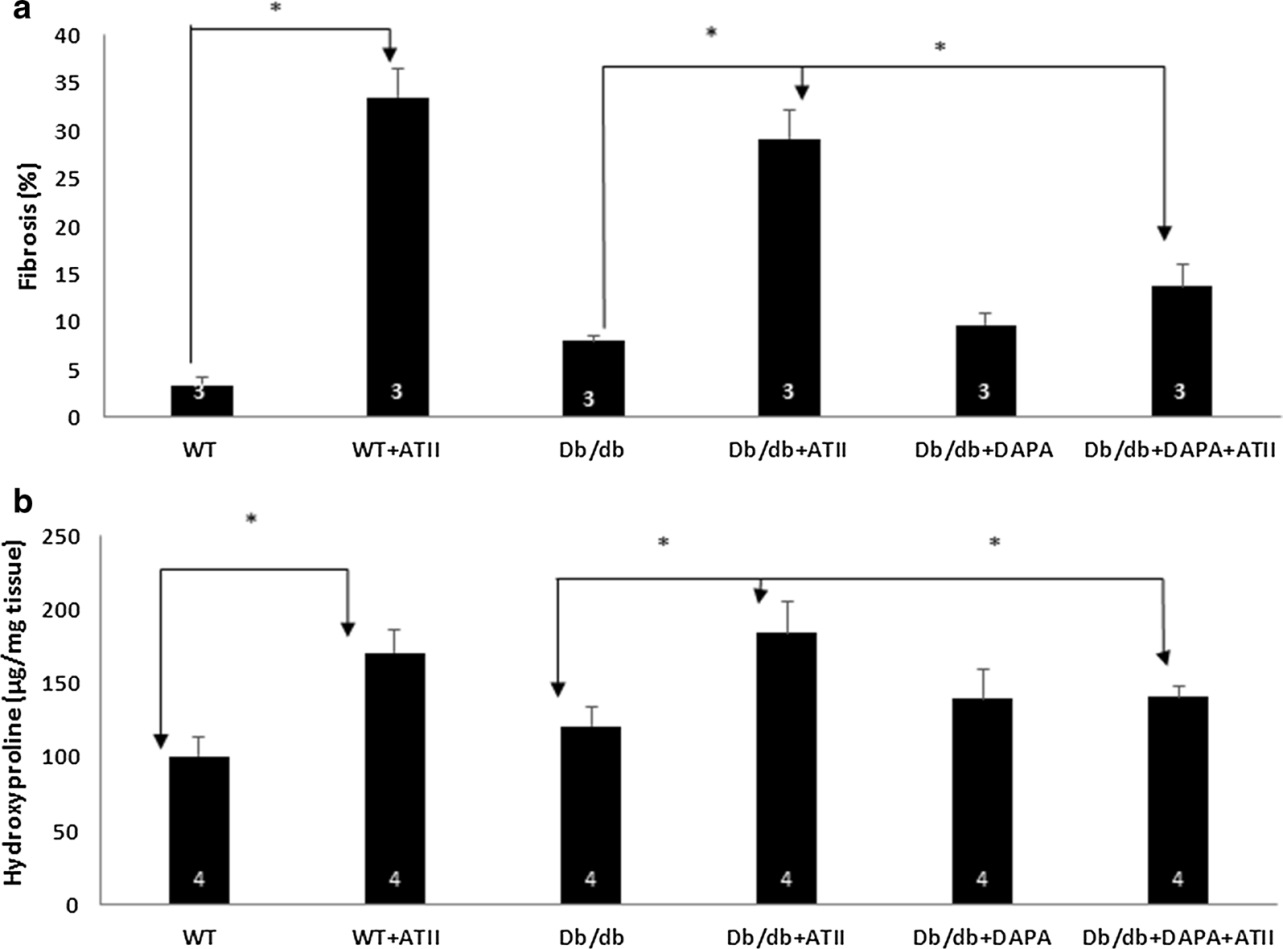
**a** Quantification of fibrosis by Photoshop illustrates the significant reduction of fibrosis by DAPA. **b** Collagen content: the hydroxyproline assay was used. Measurement of hydroxyproline has been used to quantify collagen levels. ATII increases the collagen content in both wt/wt and db/db tissues (p < 0.05) while DAPA reduced it

**Fig. 3:**
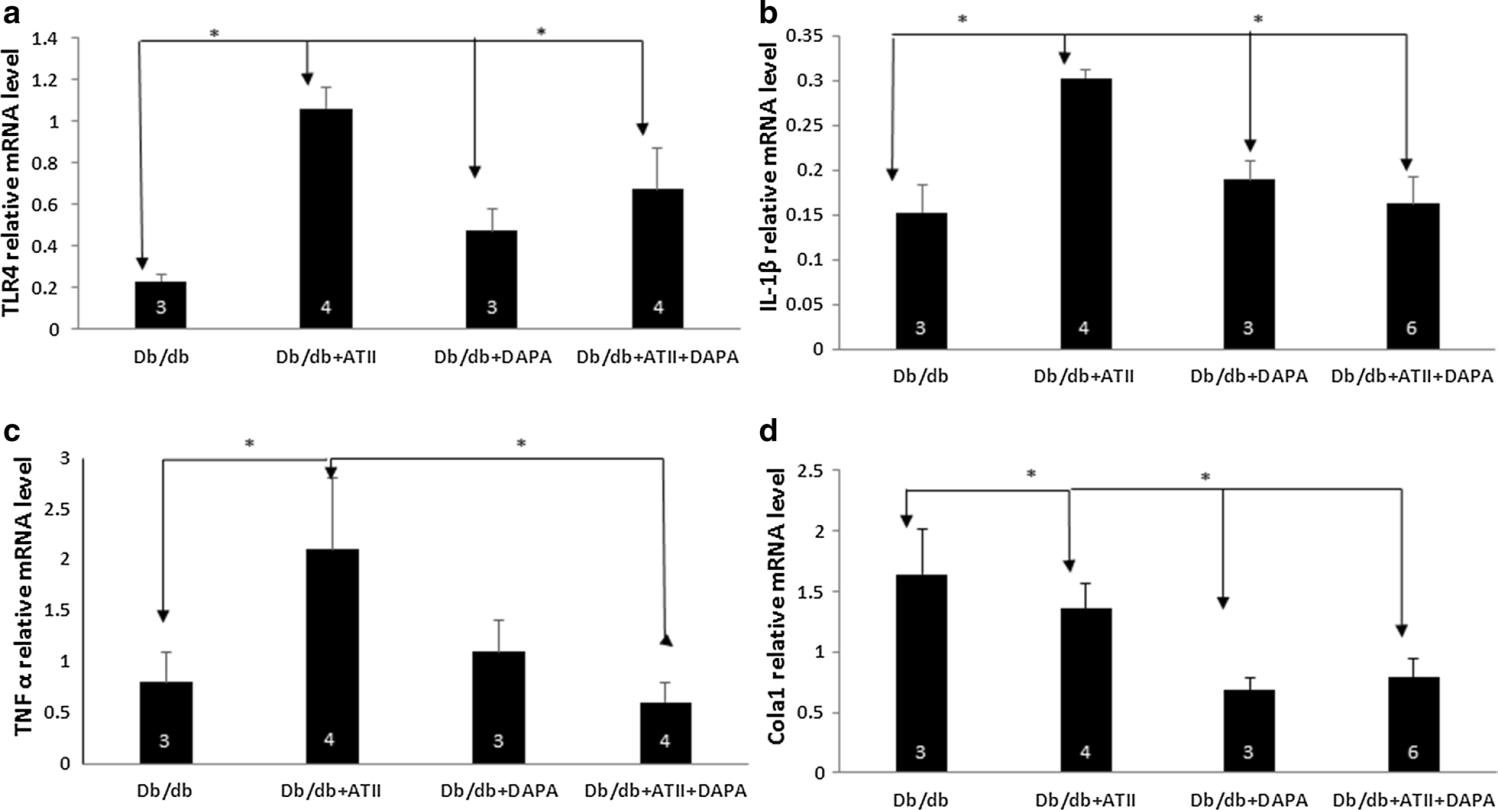
PCR analysis revealed that treating db/db mice with ATII increases the inflammatory markers TLR4 and TNFα (**a** and **c**). Adding DAPA results in a significant reduction in TLR4, IL-1β and TNFα (**a**–**c**). PCR analysis revealed that treating db/db mice with ATII increases the fibrosis marker Chola 1. However, adding DAPA causes a significant reduction in Chola 1 (**d**), (*p < 0.05)

Histopathological heart sections (hematoxylin and eosin staining) showed inflammatory cell infiltration in both wt/wt and db/db mice tissues treated with ATII (Fig. 1). However, a normal architecture was observed heart tissues of mice treated with DAPA (e and f). Masson Trichome staining showed that diabetic mice had more fibrosis than WT mice and that ATII induced the development of fibrosis in both WT and db/db mice (Fig. 1). DAPA reduced the amount of fibrotic tissue in ATII-treated db/db mice (Fig. 2a). We then used hydroxyproline assay to confirm these results by objectively quantifying the collagen content (Fig. 2b). Indeed, while ATII increases the collagen content in both wt/wt and db/db hearts (p < 0.05), DAPA tends to reduce it in ATII treated diabetic hearts. Myocardial fibrosis is characterized by accumulation of activated fibroblasts and excessive deposition of fibrotic extracellular matrix proteins, especially type I collagen. We therefore studied Cola1 mRNA expression with RT-PCR (Fig. 3). In diabetic mice, DAPA significantly decreased the levels of collagen 1 expression (Fig. 3d, p < 0.05).

Inflammatory markers were elevated in diabetic mice treated with ATII as we have previously shown [16, 17]. TLR4, TNFα and IL-1β were significantly (p < 0.05) increased in db/db + ATII compared with unstressed db/db animals (Fig. 3). Treating with DAPA had a significant effect on inflammation in db/db + ATII mice but not in unstressed diabetic mice (Fig. 3).

Cumulatively we conclude that diabetic heart is highly susceptible to ATII stress inducing changes in cardiac structure such as myocardial hypertrophy and fibrosis. The effect of Dapagliflozin was manifest in the ATII-stressed diabetic heart reducing myocardial fibrosis and improving the systolic function.

#### Investigating the mechanism of Dapagliflozin cardioprotection

**Fig. 4:**
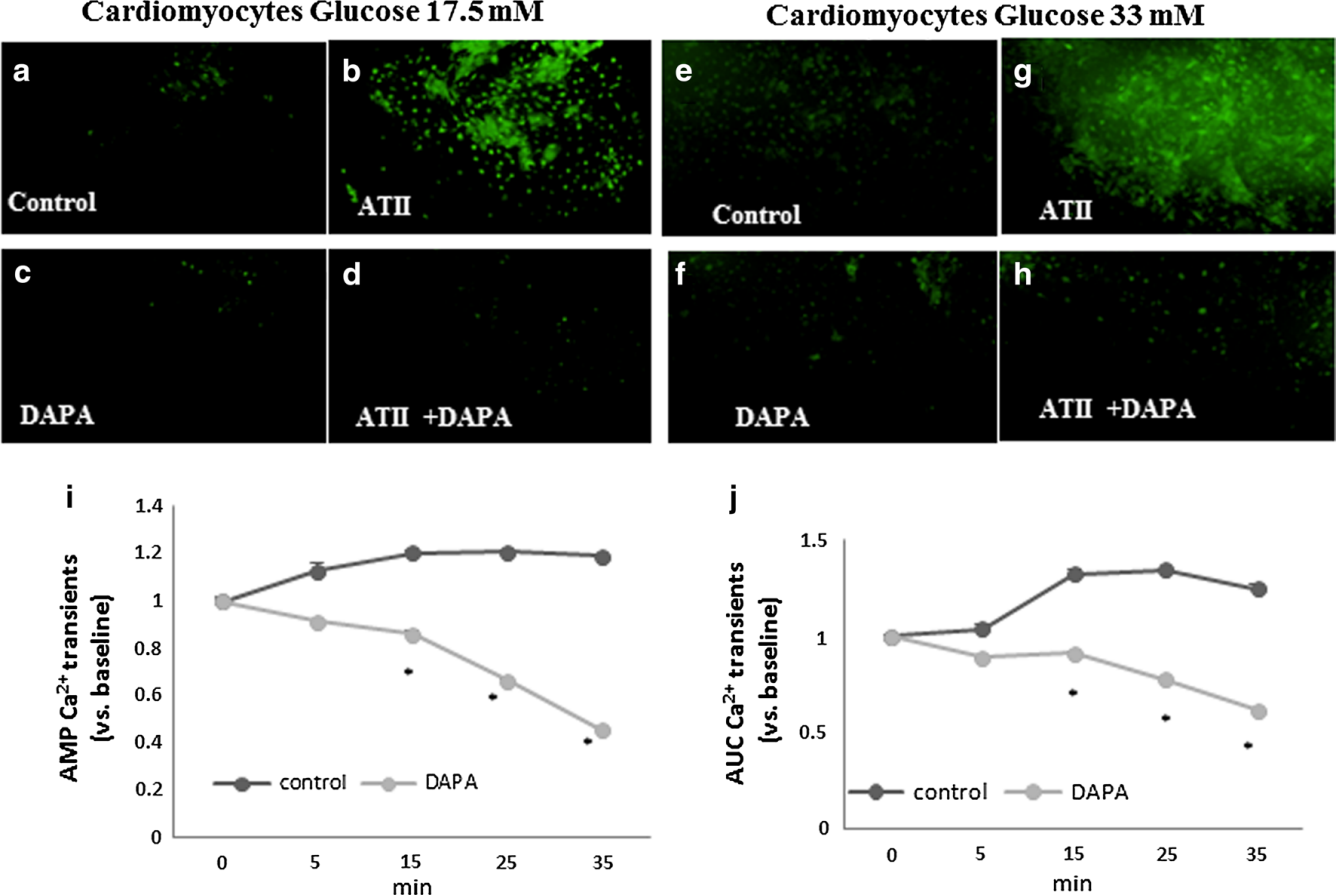
DAPA alleviates oxidative stress. Neonatal cardiomyocytes were exposed to 17.5 mM and 33 mM glucose (**a**–**h**). ROS was detected using a 2′, 7′-dichlorofluorescin diacetate (DCF-DA) reagent. Adding DAPA (5 μM), with/without ATII (1 μM) reduced the production of oxidative species in both glucose concentrations. **i**, **j** Adding DAPA to cardiomyocytes causes an immediate reduction in both amplitude (AMP) and area under the curve (AUC) of calcium transients. *p < 0.05

These experiments were performed in cardiomyocyte culture to address the direct effect of the drug on cardiac cells in isolation. Oxidative stress plays a pivotal role in the development of diabetes complications, both microvascular and cardiovascular [20]. The metabolic abnormalities of diabetes cause mitochondrial superoxide overproduction in endothelial cells of both large and small vessels, and also in the myocardium. In order to examine the interaction between DAPA glucose level and oxidative stress in the heart we used cultured rat neonatal cardiomyocytes exposed to different concentration of glucose (17.5 mM and 33 mM). Elevation of glucose levels lead to increased cellular ROS production which was further increased by ATII. Adding DAPA prior to ATII markedly reduced the oxidative stress. DAPA had an antioxidant effect on cardiomyocytes independent of glucose level (Fig. 4a–h).

To elucidate the effect of DAPA on cellular calcium, cardiomyocytes were incubated with DAPA. Adding DAPA caused a significant reduction (p < 0.05) in calcium transient amplitude (AMP) and area under the curve (AUC),*p < 0.05 (Fig. 4i, j).

#### Expression of membrane channels involved in calcium transport in heart homogenates

**Fig. 5:**
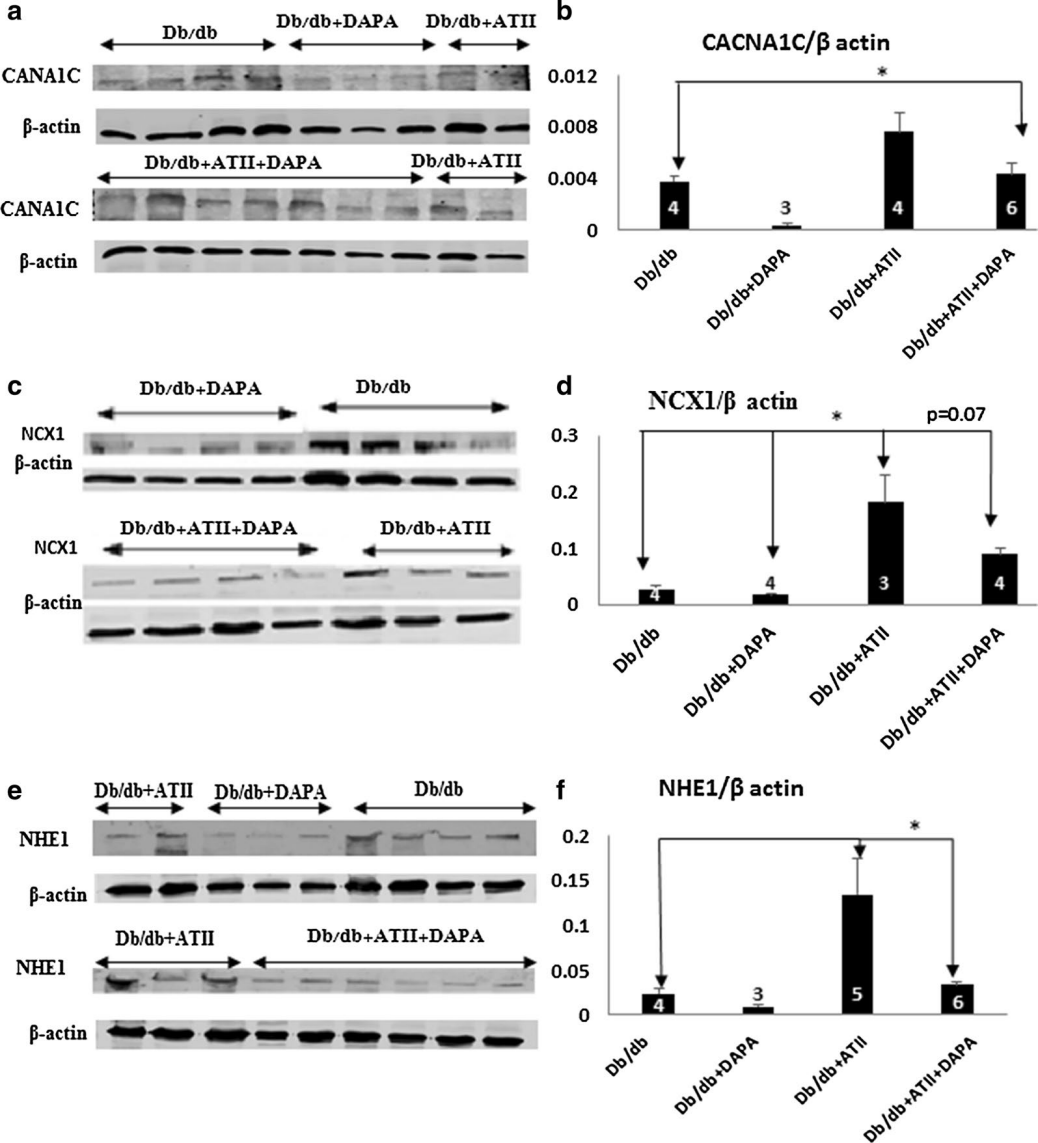
**a** Western blot for CACNA1C protein. **b** Densitometry analysis of CACNA1C normalized to β actin *p < 0.05. **c** Western blots for NCX1 protein. **d** Densitometry analysis of NCX1 normalized to β actin *p < 0.05. **e** Western blots for NHE1 protein. **f** Densitometry analysis of NHE1 normalized to β actin *p < 0.05

We measured the protein expression of voltage-dependent L-type calcium channel subunit α_1C_ (CACNA1C), NCX and NHE1. Figure 5 clearly demonstrates that while ATII increases the expression of these transporters, DAPA tends to reduce their protein levels in either untreated or ATII-stressed diabetic hearts.

Cumulatively, our in-vitro experiments suggest a mechanism where DAPA reduces calcium entry and attenuates oxidative stress associated with high glucose and angiotensin and which appears to be independent of glycemic control.

## Discussion

The significant reductions of cardiovascular events with SGLT2I’s could not be attributed solely to their glucose lowering effects. SGLT2 inhibitor (EMPA) had a protective effect in Dox-induced HF in mice as manifested by improved heart function and reduced fibrosis [21]. In the DAPA-HF trial, DAPA reduced the mortality and heart failure hospitalizations in patients with heart failure and a reduced ejection fraction with a similar benefit in those with or without diabetes [22, 23].

Studies of SGLT2 inhibitors show reductions in systolic and diastolic BP with no compensatory increase in heart rate [24]. The precise mechanism for the observed reductions in blood pressure is not fully understood but is thought to be related to SGLT2 inhibition leading to osmotic diuresis and mild natriuresis known as the diuretic hypothesis [25]. It was shown that ipragliflozin prevented LV hypertrophy and fibrosis in non-diabetic DS/obese and SHR rats without affecting plasma glucose levels [26, 27]. DAPA treatment decreases hypertension caused by ATII, and reverses LV concentric remodeling in HFpEF pigs partly by restraining sympathetic tone in the aorta, leading to inhibition of the inflammatory response [28]. DAPA was also shown effective cardioprotection in cardiac I/R injury of obese insulin-resistant rats as shown by lower infarct size and improved cardiac mitochondrial functions [29]. Another SGLT2I, Empagliflozin, also ameliorates adverse cardiac remodeling following coronary occlusion and heart failure in a nondiabetic porcine model. Empagliflozin also reduces glucotoxicity and thereby prevents the development of endothelial dysfunction, reduces oxidative stress and exhibits anti-inflammatory effects in ZDF rats, despite persisting hyperlipidemia and hyperinsulinemia [30]. Empagliflozin treatment of ob/ob mice improved coronary microvascular function and contractile performance, two measures with strong predictive values in humans for CV outcome [31]. It switches myocardial fuel utilization away from glucose toward ketone bodies, fatty acid and branched-chain amino acid, thereby improving myocardial energetics, enhancing LV systolic function, and ameliorating adverse LV remodeling [32].

In our model adding ATII to diabetes resulted in cardiomyopathy which was not found in unstressed db/db hearts [16, 17]. Diabetic heart is excessively susceptible to adverse effects of ATII [2]. Inflammation is considered an essential driving source of cardiovascular disease in diabetes. Inflammation leads to fibrosis, cell death and cardiac remodeling. Fibrosis and hypertrophy mediate diastolic stiffness which is a hallmark of the diabetic heart. We have previously shown that decreasing ROS production in a diabetes model alleviated cardiomyopathy [16]. Decreasing ROS (Fig. 4), fibrosis (Fig. 3) and inflammation (Fig. 2) what may prove to be relevant mechanism in the prevention and protection from diabetes-associated heart failure.

Our current study provides new insights into the activity of SGLT2I DAPA in the diabetic heart. DAPA showed a reduced blood pressure in ATII treated mice with a concomitant reduction blood glucose levels (Table 1). The data from the in vivo part of our study demonstrates that treatment with DAPA lowered glucose level, blood pressure, improved heart function concomitant with reduced inflammation and fibrosis in ATII-stressed db/db mice (Tables 1, 2). Hematoxylin and eosin staining showed that ATII increased mononuclear inflammatory cells infiltration in the left ventricle of heart while treating mice with DAPA reduced the leukocyte infiltration (Fig. 1). Tissue inflammatory and collagen gene expression were also attenuated following 4 weeks of DAPA treatment in ATII treated animals (Figs. 1, 3).

DAPA was previously shown to have important antioxidant-like effects in MetS-rats, similar to INS-effect, affecting Zn(2+)-regulation via Zn(2+)-transporters, MMPs, and oxidative stress [33]. Empagliflozin also improved diabetic myocardial structure and function (KK-Ay mice) in association with decreased myocardial oxidative stress and amelioration of myocardial fibrosis. The postulated mechanism appeared to be inhibition of the transforming growth factor beta/Smad pathway and activation of Nrf2/ARE signaling [34].

Our in vitro results suggest a synergy between high glucose and ATII in increasing ROS. ATII induced an increase in the inflammatory markers, TNFα, IL1-β and TLR4. DAPA reduced oxidative stress and intra cellular calcium in isolated cardiomyocytes independently of glucose concentration. Attenuation of inflammation, fibrosis and oxidative stress appears to be independent of extracellular glucose levels (Fig. 4). While we cannot exclude the possibility that DAPA inhibited glucose entry into cardiomyocytes, this possibility in unlikely given the absence of SGLT2 in cardiomyocytes. Preventing ROS excess should lead to better mitochondrial function, improving the energetic status of the myocardium and potentially explaining the improved systolic function in our model (Table 2).

Hyperglycemia and diabetes result in intracellular Na^+^ and Ca^2^ loading [35]. Increased [Na^+^]c at a steady extracellular glucose concentration was recently observed in DM2 rat cardiomyocytes, which could be ascribed to elevated Na^+^/glucose cotransport through increased expression of SGLT1 [36]. Cardiac SGLT1 expression is increased in conditions of DM2 and heart failure, in both animal and humans [36, 37]. Thus, [Na^+^]c in cardiomyocytes can be increased due to increased extracellular glucose levels as well as increased SGLT1 expression. Increases in [Na^+^]c and [Ca^2+^] c induce signaling cascades that lead to dysregulation of mitochondrial homeostasis, including impaired energetics, elevated ROS production, cardiac hypertrophy and remodeling [38]. Further cumulation of intracellular calcium levels may facilitate mitochondrial permeability transition pore opening and triggering a cascade of events commencing in cell death [39].

Elevation of [Na^+^]c results in a secondary rise of [Ca^2+^] c via the Na^+^/Ca^2+^-exchanger (NCX) [39]. Voltage-dependent L-type calcium channel is responsible for inward Ca^2+^ current in cardiac myocytes and triggers calcium release from the sarcoplasmic reticulum by activating ryanodine receptor 2 (RyR2) (calcium-induced–calcium-release) [40]. Systolic depolarization and sodium entry also lead to inward calcium flow through NCX. The increase in [Na^+^]c and [Ca^2+^]c is caused by perturbed ion fluxes due to the altered activity of ion channels and/or transporters, including the sodium–calcium exchanger (NCX), the sodium–hydrogen exchanger 1 (NHE- 1), L-type calcium channel, the ryanodine receptor regulating SR-calcium release and the sodium–potassium ATPase (Na/K ATPase) [41]. Sodium hydrogen transporter (NHE1) is responsible for cellular sodium (and therefore calcium) overload following injury and metabolic stress.

The late Na^+^ and Na/hydrogen-exchanger currents and incidence and frequency of Ca^2+^ sparks were greater in the DM2 rats than in the control. SGLT2I EMPA and DAPA reduced the amplitudes of calcium transients and currents, suggesting an alteration in the mechanism(s) of calcium transport may contribute to their cardioprotective effect [42]. The pathway involving the SGLT2I-effects on calcium transport remains unclear at the moment [19, 38]. In our study the reduction in intracellular calcium appears to be driven by a direct effect of DAPA on the expression of ion channels in the heart i.e. NHE1, NCX and L-type calcium channel.

The reduction in [Ca^2+^]c by DAPA may be related to the lowering of [Na^+^]c. Assuming no SGLT2 receptor on the sarcolemma, the [Na^+^]c lowering effects of SGLT2i can be explained by inhibition of NHE-1. Cardiomyocytes express high amounts of NHE-1, which is also of paramount importance for pH regulation during pathological conditions, including diabetes, IR injury and heart failure [38, 43]. Using freshly isolated murine ventricular cardiomyocytes and a molecular docking approach, SGLT2i Empagliflozin, Dapagliflozin, and Canagliflozin were reported to directly inhibit cardiac NHE flux and reduce intracellular sodium concentration, potentially by binding with the Na^+^-binding site of NHE-1 [38].

A note of caution is due regarding extrapolation of these results. Despite these mechanistic insights linking intracellular calcium to cardiac dysfunction, the use of both NHE-1 inhibition and calcium channel blockers proved detrimental and should be avoided in patients with heart failure with reduced ejection fraction (HFrEF) [44]. DAPA did not attenuate ATII-induced left ventricular hypertrophy in diabetes. The improving systolic function seen in DAPA-treated mice (in part explained by reduced afterload, Tables 1, 2) is of unclear consequences. The effect of SGLT2I in heart failure with preserved ejection fraction is unclear at the moment. We postulate that a unique mechanism of activity might lead to a different outcome with DAPA [11] compared to other cardiovascular drugs.

## Conclusion

**Fig. 6:**
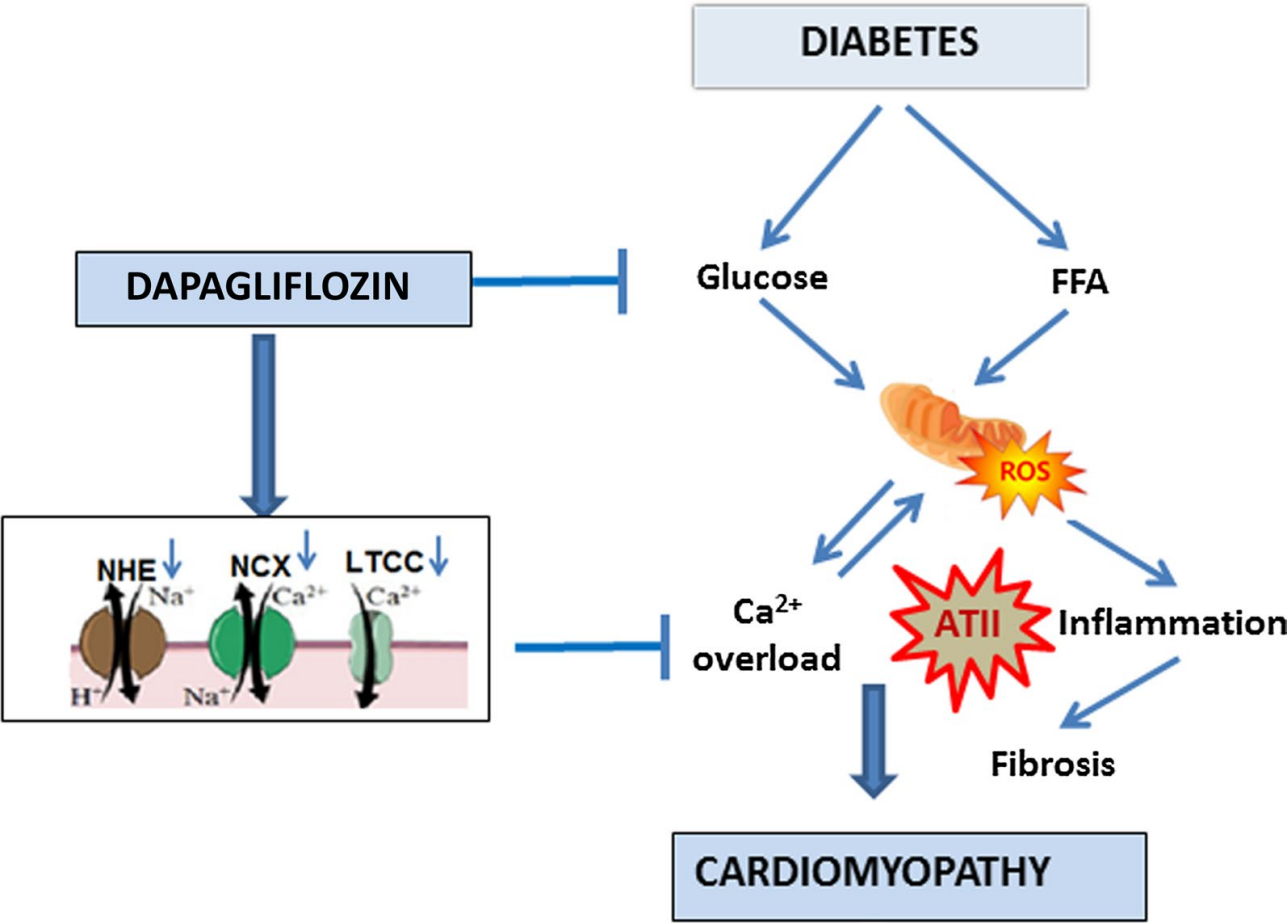
Summary of our understanding of the pathways accounting to improved cardiac function in diabetes in response to DAPA. The glucose lowering kidney targeted agent, DAPA, demonstrates direct cardiac effects by lowering myocardial [Na^+^]c through inhibition of NHE-1 and NCX and as well as Ca^2+^c through LTCC. The reduction in Ca^2+^ by DAPA appears to be directly correlated to the lowering of [Na^+^]c. The reduction in Ca^2+^ current by DAPA may partly explain the negative inotropic effects of DAPA in diabetic heart as seen by decreased hypertrophy better diastolic diameter following ATII treatment in one hand but augmented cell viability better mitochondrial function resulting less ROS production, reduced fibrosis and inflammation; all leading to better myocardial function on one hand and less cardiomyopathy markers on the other hand

Our understanding of the pathways accounting to improved cardiac function in DAPA treated hearts is illustrated in Fig. 6. We suggest that DAPA reduced intracellular calcium overload thereby reducing ROS production in the diabetic model of cardiomyopathy. Protecting the mitochondria, attenuating inflammation and preventing fibrosis would be consecutive to reducing ROS. Since all the pathological pathways in the diabetic heart are augmented in the presence of angiotensin, the effect of DAPA should be most pronounced in ATII stressed diabetic heart. Our preclinical observations provide novel insights into the possible mechanisms by which DAPA reduces cardiovascular mortality in humans.

## Data Availability

All data and materials are available upon request.

## Abbreviations

ATII: angiotensin II
AUC: area under the curve
AMP: amplitude
[Ca^2+^]c: cytosolic calcium
CACNA1C: voltage-dependent L-type calcium channel subunit α_1C_
db/db mice: diabetic mice with leptin deficiency
DM2: Diabetes Mellitus Type 2
DAPA: Dapagliflozin
EF: ejection fraction
FS: fractional shortening
H&E: hematoxylin and eosin
HO-1: heme oxygenase-1
IL-1β: interleukin 1β
IVSd and IVSs: interventricular septal end diastole and end systole diameter
LV: left ventricle
LVIDd and LVIDs: left ventricular internal end diastole and end systole diameter
LVPWd and LVPWs: left ventricular posterior wall end diastole and end systole diameter
[Na^+^] c: cytosolic sodium
NCX: the sodium–calcium exchanger
NHE-1: the sodium–hydrogen exchanger 1
SGLT2I: sodium glucose transporter 2 inhibitor
ROS: reactive oxygen species
TLR4: toll like receptor 4
TNFα: tumor necrosis factor α
WT: wild type
